# Viral Hepatitis in Indonesia: Past, Present, and Future

**DOI:** 10.5005/jp-journals-10018-1171

**Published:** 2016-07-09

**Authors:** Mulyanto

**Affiliations:** Faculty of Medicine, West Nusa Tenggara Hepatitis Laboratory, Mataram and Immunobiology Laboratory, University of Mataram, Mataram, West Nusa Tenggara, Indonesia

**Keywords:** Epidemiology, HBsAg, HCV RNA, Indonesia.

## Abstract

Since Indonesia is a huge archipelago country, the prevalence of hepatitis virus infection highly varies among islands. In average the prevalence of clinical hepatitis in Indonesia was 0.6% in the year 2007. Of 82 clinical acute hepatitis patients treated in hospitals in several cities, acute hepatitis A accounted for 28.0%, acute hepatitis B 13.4%, and acute hepatitis C 1.2%; 35.4% patients were infected by unknown etiology. In 1980s, the prevalence of anti-HAV in the age group 10- to 14-years was almost 100% in smaller towns and 45 to 60% in big cities. About 30 years later, anti-HAV prevalence decreased to around 13% in the same smaller town. Outbreaks of hepatitis A were reported between 2006 and 2009 in several cities in Java island. The prevalence of HBsAg in the islands other than Java island (8.5%) was significantly higher than in Java island (4.9%). In 1,409 viremic subjects, 4 genotypes of HBV were found, i.e., genotype B (60%), followed by genotype C (33%), genotype D (7%), and genotype A (0.3%). In contrast, the prevalence of hepatitis C was much higher in Java island compared to other islands. In blood donors in Java island, the prevalence of anti-HCV and HCV RNA were 1.5 and 1.1% respectively, while in other islands, the prevalence were only 0.7 and 0.2% respectively. Hepatitis D was very rare in Indonesia, and so was hepatitis E. Outbreaks of hepatitis E were reported between 1989 and 1993 in West Kalimantan. Afterward, the incidence of hepatitis E was reported sporadically.

**How to cite this article:**

Mulyanto. Viral Hepatitis in Indonesia: Past, Present, and Future. Euroasian J Hepato-Gastroenterol 2016;6(1):65-69.

## INRODUCTION

Indonesia is an archipelago country consisting over 13,000 islands, inhabited by more than 650 tribes, with each tribe having its own ethnic group language. The hygiene and sanitation conditions are highly variable between islands. Ethnic variations are mainly found in the eastern part of Indonesia, ranging from East Nusa Tenggara up to Papua. The population of Indonesia in 2014 was around 254,455,000 with an income of USD 3,491.9 per capita.^1^ Based on the Riskesdas (Basic Health Research) in 2007, the prevalence of clinical hepatitis in Indonesia was between 0.2 and 0.9% with an average of 0.6%. In the age group 1 to 4 years, 5 to 14 years, and 35 to 44 years, the prevalence was 0.3, 0.4, and 0.7 respectively.^2^ Patients with acute hepatitis treated in hospitals in several cities in Indonesia mostly suffer from acute hepatitis A. Mulyanto et al^3^ reported that of 82 patients with acute hepatitis in 2007 in Solo (Central Java), Denpasar (Bali island), Mataram (Lombok island), and Makassar (Sulawesi island), both the HAV-RNA and anti-HAV IgM were found positive in 23 patients (28.0%).

## HEPATITIS A

The prevalence of anti-HAV in Indonesia is generally very high, and the prevalence in the population in small towns is much higher than in the big cities. Almost 100% of children in the age group 10 to 14 years in Jayapura (Papua), Mataram (Lombok island), and Sumbawa Besar (Sumbawa island) were infected with HAV. However, in the same age group it was reported that in Jakarta and Bandung (Java) and Makassar only around 45 to 60% of children had been infected with HAV.^4^ In addition to the tendency of lower anti-HAV prevalence in big cities with hygienic and sanitary conditions relatively better, there was a trend toward a decrease in the prevalence of anti-HAV in line with time. Lately, it was reported that the prevalence of anti-HAV was much lower than that in the era before the 1990s. In 2006, among 300 adult subjects (18–19 years) in Jayapura and Biak, the prevalence of anti-HAV was 60%. In the indigenous population, the prevalence was much higher than that in the nonindigenous population, i.e., 81 *vs* 50% (Mulyanto, unpublished data). This may be due to the fact that the indigenous people live in areas with poor hygiene and sanitation conditions compared to the nonindigenous Papuans. Furthermore, Gunawan et al^5^ reported that almost 30 years later in Mataram, among 110 students aged 12 to 14 years, it was found that only 15 (13.64%) individuals were anti-HAV positive; of the 108 subjects from the age group 16 to 20 years, only 70 (64.8%) were found to be anti-HAV positive.

The lower prevalence of anti-HAV among children on the island of Java had caused more frequent hepatitis A outbreaks. It had been reported that the outbreak of hepatitis A occurred consecutively in Bogor (West Java) in 1998; Jember and Bondowoso (East Java) in 2006; Tangerang (suburb of Jakarta) in 2007; Yogyakarta in 2008; and Ngawi (East Java) in 2009, in which a case of icteric in each area accounted for as many as 74, 50, 17, 1,160, and 146 people respectively (Sub-directorate of Surveillance and Outbreak Response, Directorate General of Disease Control and Environmental Health, Ministry of Health, personal communication). Among 82 samples with clinical acute hepatitis treated in several cities in Indonesia, i.e., in 2003 in Denpasar (n = 38), and in 2007 in Solo (n = 19), Mataram (n = 17), as well as Makassar (n = 8), acute hepatitis A accounted for 28.0%, acute hepatitis B 13.4%, and acute hepatitis C 1.2%, while 35.4% had infections of unknown etiology (non-A to E). Both acute hepatitis D and acute hepatitis E were not found. A total of 34 sera samples consisting 11 individuals during outbreak of hepatitis A in Jember and Tangerang, and 23 samples with acute hepatitis A in Solo, Denpasar, Mataram, as well as in Makassar, were examined for HAV genotyping. Genomic sequences analysis confirmed that the 34 samples formed a single phylogenic cluster of subgenotype IA, and classified as Indonesian IA sublineage.^3^

## HEPATITIS B

Based on the prevalence of HBsAg, Indonesia is cate-gorized as hepatitis B endemic country with intermediate-to-high endemicity; the prevalence varies greatly across regions and islands. In general, the prevalence of HBsAg in islands outside of Java was significantly higher than in Java. Among 1,000 blood donors collected in Jakarta in 1989, the prevalence of HBsAg (R-PHA test) was 4.9%. While in the eastern part of Indonesia, among 5,117 blood donors collected in 1995 and 4,047 in 2006/2007, the prevalence of HBsAg (R-PHA test) was found between 4.2 and 15.8% with an average of 8.5%. A very high HBsAg prevalence was found in some areas in the eastern part of Indonesia, such as in Mataram (10%), Kupang, Timor island (15.8%), Ambon, Moluccas (9.1%), and Sorong, Papua (17%).^6^ However, there was also a low prevalence of HBsAg in some villages in the eastern part of Indonesia, such as in the village of Enggros (3.1%) and Tarfia (4.1%), in the district of Jayapura, Papua.^7^ In accordance with its endemicity, transmission of HBV usually occurs vertically from hepatitis B suffering mother to her child at birth and horizontally to children of early age. Various attempts have been made to fight against hepatitis B, which include mass hepatitis B vaccination and the treatment of HBV-infected patients. Although HBV infection remains a major health problem in adults, it seems that HB immunization has begun to decrease the prevalence of HBV infection in the younger generation in Indonesia.

Furthermore, the biodiversity of Indonesian population also affects molecular variation of HBV. Of the 4 existing HBsAg subtypes, all can be found in Indonesia. In the western part of Indonesia, from Sumatra to Kalimantan and the island of Sumbawa, adw subtype is predominant. While in East Nusa Tenggara and Moluccas, ayw subtype is found predominant. In Papua, adr subtype is the predominant serotype. Ayr subtype is very few and hardly found in several regions in Indonesia, such as Jakarta, the village of Bayan, Lombok, and Manado, North Sulawesi.^8^ To date, 10 HBV genotypes have been reported worldwide, i.e., genotype A-J, and 42 subgenotypes. At least 4 genotypes among them are found in Indonesia, i.e., genotypes A, B, C, and D, although genotype A was infrequent. From more than 1,400 viremic subjects in 33 cities/villages on 17 islands covering nearly all major islands of Indonesia, HBV was segregated into 4 genotypes: Genotype B was predominant (60%), followed by genotypes C (33%), D (7%), and A (0.3%).^7-11^

**Fig. 1:**
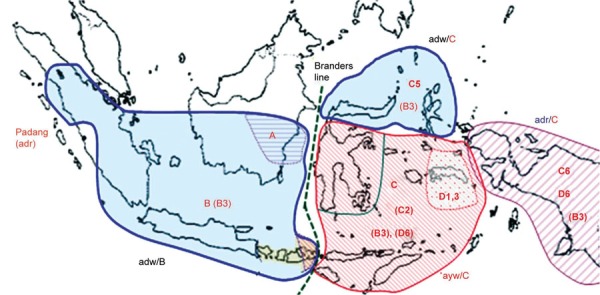
Map of Indonesian archipelago showing four major HBV serotype/subgenotype zones. The four zones reflecting the distinct geographical distribution of HBV serotypes/subgenotypes in Indonesia are shaded. The Branders line (the line that lies from the strait between Sumbawa and Flores islands to the north between Kalimantan and Sulawesi islands) divides Indonesia to genotype B zone in the west and genotype C zone in the east

Of the 42 HBV subgenotypes, i.e., A1-6, B1-9, C1-16, D1-7, and E1-4 known in the world,^12^ 25 (59%), i.e., A1-2, B1-3, 5, 7-9, C1-2, 5-6, 8-16, and D1,3,6 were found in Indonesia; even 14 subgenotypes (B7-9; C6, C8-16; and D6) were first discovered in Indonesia.^7-10,13-15^ Among the 25 subgenotypes found in Indonesia, HBV of subgenotype B3 (HBV/B3) accounted for 34%. Hepatitis B virus/B3 was distributed widely in Indonesia, and has not been reported in countries other than Indonesia. Hence, it is very likely that HBV/B3 is indigenous to Indonesia.^8^ A recent study showed that HBV serotypes/subgenotypes distribution in Indonesia can be divided into 4 zones (Fig. 1), namely adw/B3 zone from Sumatra to Sumbawa and southern part of Sulawesi, adw/C5 in northern part of Sulawesi and North Moluccas, ayw/C2 zone in the region of East Nusa Tenggara and South Moluccas, and adr/C6 zone in Papua Indonesia.^11^ Studies on mice indicated that the immune response against the epitope “d/y” usually appears 1 to 2 weeks earlier than that of antigenic determinant “a”.^16^ Hence, under a certain condition where rapid immune response is needed, such as the vaccination of babies born to HBV-infected mothers, it may be suggested that in the future, recombinant hepatitis B vaccines that will be used in Indonesia ideally should be derived from the 4 HBV serotype/genotype genomes.

Our findings enriched the knowledge about the distribution of HBsAg serotypes and HBV genotypes/subgenotypes, which indicates that the spread of HBsAg subtypes and HBV genotypes/subgenotypes is extremely ethnic/region-dependent in Indonesia, a country that has a motto “Bhineka Tunggal Ika,” or unity in diversity. The steady existence of the B3 subgenotype found in our study indicates that although Indonesia is a country with diversity of HBV subgenotypes discovered; B3 can reunite all and it reflects the motto of the country.^11^

## HEPATITIS C

In general, the prevalence of anti-HCV in Java is higher than outside Java. Conversely, the prevalence of HBsAg outside Java is higher than in Java. In 1995 among 6,971 blood donors in 4 big cities in Java, i.e., Jakarta, Bandung, Solo, and Surabaya, the prevalence of anti-HCV and HCV RNA were 1.5 and 1.1% respectively. While from 8,183 blood donors in 10 big cities outside Java, i.e., Medan, Palembang, and Padang (Sumatra island), Banjarmasin and Pontianak (Kalimantan), Manado and Makassar (Sulawesi island), Kupang and Dili (Timor island), and Ambon (Moluccas), the prevalence of anti-HCV and HCV RNA were 0.7 and 0.2% respectively.^17^ Furthermore, of the 44 viremic blood donors in Jakarta, Bandung, Solo, and Surabaya, HCV-1 and HCV-2 accounted for 68 and 32% respectively,^17^ and of 52 patients with chronic liver disease (CLD), in Surabaya, 67% were HCV-1 and 13% were HCV-2.^18^

The high prevalence of HCV among blood donors is also reflected in the higher prevalence of anti-HCV in patients with CLD in big cities in Java than in those of big cities outside Java. In 1992, the prevalence of anti-HCV in patients with CLD (chronic hepatitis and liver cirrhosis) in Surabaya, Java island, was much higher than the prevalence in Mataram. Of the 343 patients with CLD in Dr. Soetomo General Hospital, Surabaya, and 114 patients with CLD in Mataram General Hospital, the prevalence of anti-HCV was much higher in Surabaya (61.8%) compared to those in Mataram (14.9%); conversely, HBsAg positives were only 27.7% in Surabaya and 41.2% in Mataram. Similarly, the prevalence of non-B-non-C hepatitis virus (NBNC) was much higher in patients with CLD in Mataram (43.9%) compared to those in Surabaya (9.6%). One of the possible reasons of high prevalence of NBNC in patients with CLD in Mataram was due to the existence of aflatoxin-contaminated food in the past.^17^

## HEPATITIS D

Hepatitis D virus is very rare in Indonesia. It has been observed that of 94 HBsAg-positive of indigenous Papuans in Jayapura in 2006, there were 2 anti-HDV IgG positives found but no HDV-RNA was found in both subjects (Mulyanto, unpublished data). Among 63 patients with acute hepatitis from Denpasar, Mataram, and Makassar and 95 subjects whose serum samples were obtained for screening during outbreaks of hepatitis A in Indonesia in 2006 and 2007, all patients/subjects were negative for anti-HDV IgG.^3^

## HEPATITIS E

There are not many reports on HEV infection in Indonesia. It was reported that in 1989–1993, the hepatitis E outbreak occurred in West Kalimantan, where people commonly use river water for daily activities.^4^ In 2006, among 756 subjects 18 to 19 years of age in Jayapura and Biak, the prevalence of anti-HEV IgM/IgA was 7.4%. The prevalence in indigenous population (13%) was greater than in nonindigenous population, which was only 6% (Mulyanto, unpublished data). Among 63 patients with acute hepatitis from Denpasar, Mataram, and Makassar and 95 subjects whose serum samples were obtained for screening during outbreaks of hepatitis A in Indonesia in 2006 and 2007, all patients/subjects were negative for anti-HEV IgM/IgA.^3^

## PREVENTION

Various attempts have been made to fight against hepatitis B and hepatitis C, which include HBsAg screening among blood donors, mass hepatitis B immunization, and the treatment of HBV-infected patients. To date no mass hepatitis A immunization has been done in Indonesia. HBsAg screening among blood donors in Indonesia has been compulsory since 1996; meanwhile, anti-HCV screening has been compulsory since couple years later. In general, screening was done by using ELISA and in some Blood Transfusion Units by using the IC method. However, in some big cities like Jakarta, Bandung, and Semarang, some donors were screened by using NAT. Besides HBsAg screening among blood donors, nationwide HBV immunization has been integrated to National EPI since 1997, which was initiated by the Hepatitis B Model Immunization Project in Lombok in 1987. The project was of 4 years duration and started with 18 villages excluding Mataram city. After 4 years, the project was able to show 77% decrease (from 6.2% before starting the project to 1.9% in the final year of the project) in HBsAg prevalence among less than 4 year-old children in the study villages. Some data collected and evaluation indicated that Nationwide Hepatitis B Immunization was successful in reducing the prevalence of HBsAg by approximately 80% of the children population who received vaccination. Whereas a study conducted in Mataram showed that there was a decrease in the prevalence of HBsAg in the 1 to 4 year-old children from 4.1% before vaccination to 1.7% in 1999 and to 1.1% in 2012.^19,20^

## DIAGNOSIS AND TREATMENT

Besides AST and ALT tests, serological hepatitis virus markers, such as IgM anti-HAV, HBsAg, HBeAg/anti-HBe, IgM anti-HBc, and anti-HCV are also applied as a routine test, as well as abdominal ultrasonographic examination. Quantitative HBV DNA and HCV RNA tests are done prior to treatment of CLD patients. Nationwide, the treatments of viral hepatitis are done according to “The Guidance of Treatment of Chronic Liver Disease” issued by the Indonesian Association for the Study of the Liver. The antiviral drugs used for the treatment of hepatitis B are interferon alfa, pegylated interferon, and nucleosides analogs such as lamivudine, adefovir dipivoxil, entecavir, and telbifudine, whereas those for the treatment of hepatitis C are pegylated interferon, ribavirin, and sofosbuvir. One obstacle in the treatment of patients with chronic hepatitis B is the high price of drugs and diagnostics. With the nucleoside analogue drugs and diagnostics produced in the country, more and more patients can be treated.

## CONCLUSION

The prevalence of hepatitis A in Indonesia has decreased in the last 30 years, in accordance with the improving hygienic and sanitation conditions. The lower prevalence of anti-HAV among children makes possible for hepatitis A outbreak. Although hepatitis B still exists as a serious public health problem in adults, hepatitis B immunization has decreased the prevalence to around 20% as compared to before immunization. Hence, one generation later, prevalence of hepatitis B is presumed to show a number of 1 to 2%. The prevalence of hepatitis C in big cities in Java is much higher compared to that in the cities/areas outer Java. However, by doing anti-HCV screening on blood donors nationwide, it is presumed at least the prevalence will not increase anymore. Hepatitis D is not a public health problem, since practically HDV is not found in Indonesia. Outbreaks of hepatitis E were recorded in 1989–1993 in Kalimantan. Hepatitis E is found only sporadically in some areas in Indonesia.
